# Exscalate4CoV: Innovative High Performing Computing (HPC) Strategies to Tackle Pandemic Crisis

**DOI:** 10.3390/ijms231911576

**Published:** 2022-09-30

**Authors:** Andrea R. Beccari, Giulio Vistoli

**Affiliations:** 1 EXSCALATE, Dompé Farmaceutici S.p.A., Via Tommaso De Amicis 95, I-80131 Napoli, Italy; 2 Dipartimento di Scienze Farmaceutiche, Università degli Studi di Milano, Via Mangiagalli 25, I-20133 Milano, Italy

This Special Issue was intended as a dissemination forum where the major results pursued by the EXSCALATE4CoV project (E4C, https://www.exscalate4cov.eu, accessed on 1 September 2022) could be exhaustively reported to enable the employment of the shared data for novel independent studies. The EXSCALATE4CoV (E4C) project aimed to exploit the most powerful computing resources currently based in Europe to support the rapid identification of new treatments against SARS-CoV-2. Advanced computer-aided drug designs (CADDs) were combined with high-throughput biochemical and phenotypic screening studies to accelerate the discovery of new drugs. In a pandemic crisis, the rapid identification of effective treatments has a dramatic relevance. E4C was mostly based on the already developed and validated EXSCALATE docking platform (https://www.exscalate.eu/en/platform.html, accessed on 1 September 2022) and its activities were empowered by three of the most powerful computer centers in Europe: CINECA, BSC, and JÜLICH.

In detail, this issue includes four research articles plus a review. The first original study reports an exhaustive profiling of the druggable binding sites for the therapeutically relevant SARS-CoV-2 proteins [1]. This study represents the necessary starting point for all of the following docking simulations and virtual screening campaigns. The binding sites are characterized by using a purposely developed tool, implemented into the VEGA suite of programs [2], which combines the pocket search as performed by the FPocket software [3] with docking simulations of representative ligands to more effectively investigate the interactive potential of the explored cavities. The so-defined binding sites are then utilized by the Mediate initiative as described below. The paper by Grottesi and co-workers [4] describes microsecond time scale MD simulations focused on the dimeric structure of 3CL-Pro in its apo state. The analysis of the dynamic profile of the simulated homodimer highlights the key role played by the conformational shifts exhibited by the loop that modulates the accessibility of the binding pocket, thus playing a key role in determining the catalytic activity and the interactions of potential inhibitors. The key role of small conformational changes of the regions surrounding the catalytic dyad in determining the inhibitory activity towards 3CL-Pro was then assessed by Rossetti and co-workers by combining molecular docking, MD simulations, and pharmacophore mapping [5]. While considering the marked flexibility of the 3CL-Pro binding site, structure-based virtual screening campaigns afforded satisfactory performances, especially when combining the results from different docking engines [6].

The second group of original studies, authored by Chillemi and co-workers, focuses on the Spike glycoprotein. The first study reports an evolutionary analysis of various Spike glycoproteins by combining phylogenetic recombination detection and natural selection analyses with MD simulations [7]. The obtained results evidenced that the considered Spike variants, while showing significant structural differences, retain two main long-range covariant dynamic movements that involve the receptor-binding domain, the PRRA and KR*SF cleavage sites, and conserved regions in the terminal domains. These results suggest that selection increases the structural variability and flexibility of the Spike glycoproteins but preserves some key inter-domain fluctuations that are essential for its activity and could be exploited for the rational design of efficient inhibitors. The second study investigates the structural effects of the mutations found in the Spike glycoprotein of the SARS-CoV-2 Variant of Concern 202012/01 (B.1.1.7, i.e., the UK variant) by performing microsecond-long MD simulations [8]. The comparison of the considered mutant with the wild-type revealed that the binding domain (RBD) of the former is characterized by a greater flexibility, a feature that can explain the increased transmission reported for this variant. Similar MD-based comparative studies also involved Delta (B.1.617.2) and Omicron (BA.1) Spike Glycoproteins [9]. A comparison with the wild type revealed that the different transmissibility of these variants might be ascribed to the greater flexibility of a small region of RBD and of the so-called “fusion-peptide proximal region”. The role of this increased flexibility is further corroborated through a consideration of the fact that the two detected regions are involved in ACE2 recognition and membrane fusion. Finally, this issue includes a review that deals with the potential entry inhibitors encompassing both small molecules and peptides targeting both viral and host proteins [10].

More generally, the E4C project achieved outstanding results, as witnessed by some relevant deliverables: (1) overall, the E4C consortium identified about 600 new active molecules by producing more than 70,000 screening data—in this context, the results from a large-scale repurposing campaign by cytopathic SARS-CoV-2 screening of VERO-E6 cells have been recently published [11]; (2) these intensive and multifaceted computational efforts culminated in the largest and fastest virtual screening simulation ever run, in which more than 1 trillion docking simulations were run within a single shoot, along with the deployment of ad hoc virtual screening protocols and the X-ray validation of the most relevant findings [12,13]; (3) a new suite of open bioinformatics and simulation tools was released to the global research community—this includes three web platforms that comprise the most complete (>60 simulations) and informative (>10 µs) sets of SARS-CoV-2 molecular dynamics simulations, which were enabled by the use the best European HPC resources; (4) finally, a clinical candidate, raloxifene, which stemmed from the repurposing efforts, completed a trial to test its safety and efficacy with the enrolment of 150 patients in three EU countries, as recently reported [14].

Among the initiatives developed within the E4C framework, Mediate (https://mediate.exscalate4cov.eu, accessed on 1 September 2022) represents the prototype of a collaborative platform to perform virtual screening and drug-repurposing campaigns. The Mediate initiative invites worldwide researchers to contribute with the results of their own simulations based on a set of protein structures and compound libraries specifically prepared and shared by the E4C project. The submitted simulations are then integrated by global consensus strategies, based on AI techniques, and can enhance the resulting predictive power. The best compounds so-identified are then purchased and tested and the biological results will be shared within the scientific community.
